# MF-Net: Multi-Scale Information Fusion Network for CNV Segmentation in Retinal OCT Images

**DOI:** 10.3389/fnins.2021.743769

**Published:** 2021-10-08

**Authors:** Qingquan Meng, Lianyu Wang, Tingting Wang, Meng Wang, Weifang Zhu, Fei Shi, Zhongyue Chen, Xinjian Chen

**Affiliations:** School of Electronics and Information Engineering, Soochow University, Jiangsu, China

**Keywords:** choroid neovascularization, OCT images, multi-scale information fusion network, segmentation, convolutional neural networks

## Abstract

Choroid neovascularization (CNV) is one of the blinding ophthalmologic diseases. It is mainly caused by new blood vessels growing in choroid and penetrating Bruch's membrane. Accurate segmentation of CNV is essential for ophthalmologists to analyze the condition of the patient and specify treatment plan. Although many deep learning-based methods have achieved promising results in many medical image segmentation tasks, CNV segmentation in retinal optical coherence tomography (OCT) images is still very challenging as the blur boundary of CNV, large morphological differences, speckle noise, and other similar diseases interference. In addition, the lack of pixel-level annotation data is also one of the factors that affect the further improvement of CNV segmentation accuracy. To improve the accuracy of CNV segmentation, a novel multi-scale information fusion network (MF-Net) based on U-Shape architecture is proposed for CNV segmentation in retinal OCT images. A novel multi-scale adaptive-aware deformation module (MAD) is designed and inserted into the top of the encoder path, aiming at guiding the model to focus on multi-scale deformation of the targets, and aggregates the contextual information. Meanwhile, to improve the ability of the network to learn to supplement low-level local high-resolution semantic information to high-level feature maps, a novel semantics-details aggregation module (SDA) between encoder and decoder is proposed. In addition, to leverage unlabeled data to further improve the CNV segmentation, a semi-supervised version of MF-Net is designed based on pseudo-label data augmentation strategy, which can leverage unlabeled data to further improve CNV segmentation accuracy. Finally, comprehensive experiments are conducted to validate the performance of the proposed MF-Net and SemiMF-Net. The experiment results show that both proposed MF-Net and SemiMF-Net outperforms other state-of-the-art algorithms.

## Introduction

Choroidal neovascularization (CNV), also known as subretinal neovascularization, is a basic pathological change of various intraocular diseases such as age-related macular degeneration, central exudative chorioretinopathy, idiopathic choroidal neovascularization, pathological myopic macular degeneration, and ocular histoplasmosis syndrome (DeWan et al., 2006; Abdelmoula et al., 2013; Jia et al., 2014; Liu et al., 2015; Zhu et al., 2017). It often involves the macula, causing serious damage to the central vision. In the early stage of CNV, there are usually no abnormal symptoms. Along with the gradual expansion of neovascular leakage and rupture, it may cause vision loss, visual distortion, or central scotoma (Freund et al., 1993; Grossniklaus and Green, 2004). CNV can persist for months or years and then gradually become steady (Zhu et al., 2017). The macula of the patients with recurrent symptoms are seriously damaged, which may cause permanent visual impairment. Optical coherence tomography (OCT) is a non-invasive imaging technology proposed by Huang et al. (1991), which can capture high-resolution cross-sectional retinal structure. It plays an important role in the diagnosis and monitoring of retinal diseases (Shi et al., 2014; Chen et al., 2015; Wang et al., 2021a). In addition, fluorescence angiography (FA) and indocyanine green angiography (ICGA) are also important diagnostic imaging modalities for the detection of retinal diseases in clinical practice, and there are many works to analyze CNV based on FA and ICGA (Talisa et al., 2015; Gao et al., 2016; Corvi et al., 2020). However, FA and ICGA can only capture one 2D fundus image, which may cause the loss of internal structure information of CNV (Zhang et al., 2019). Besides, FA and ICGA are invasive and may cause nausea and other allergic reactions due to intravenous injection of dye (Jia et al., 2014). Instead, OCT is non-invasive and can obtain high-resolution cross-sectional images of the retina with a high speed (Talisa et al., 2015; Corvi et al., 2020). Therefore, accurate segmentation of CNV in OCT images is essential for ophthalmologists to analyze the condition of the patient and specify treatment plan. There are also previous studies that have been proposed for CNV segmentation in retinal OCT images (Xi et al., 2019; Zhang et al., 2019). Zhang et al. (2019) designed a multi-scale parallel branch CNN to improve the performance of CNV segmentation in OCT images. Xi et al. (2019) proposed an automated segmentation method for CNV in OCT images using multi-scale CNN with structure prior, in which a structure learning method was innovatively proposed based on sparse representation classification and the local potential function to capture the global spatial structure and local similarity structure prior. However, CNV segmentation in retinal OCT images is still very challenging as the complicated pathological characteristics of CNV, such as blur boundary, large morphological differences, speckle noise, and other similar disease interference. Multi-scale global pyramid feature aggregation module and multi-scale adaptive-aware deformation module are proposed to segment corneal ulcer in slit-lamp image in our previous study (Wang et al., 2021b). Therefore, to tackle these challenges and improve the CNV segmentation accuracy, a novel multi-scale information fusion network (MF-Net) is proposed for CNV segmentation in retinal OCT images. Our main contributions are summarized as follows,

1) A multi-scale adaptive-aware deformation module (MAD) is used and inserted at the top of encoder path to guide the model to focus on multi-scale deformation of the targets and aggregates the contextual information.
2) To improve the ability of the network to learn to supplement low-level local high-resolution semantic information to high-level feature maps, a novel semantics-details aggregation module (SDA) between encoder and decoder is designed.
3) Based on a U-shape architecture, a novel MF-Net integrated MAD module and SDA module are proposed and applied for CNV segmentation tasks. In addition, to leverage unlabeled data to further improve the CNV segmentation accuracy, a semi-supervised version of MF-Net is proposed by combining pseudo-data augmentation strategy named as SemiMF-Net.
4) Extensive experiments are conducted to evaluate the effectiveness of the proposed method. The experimental results show that, compared to state-of-the-art CNN-based methods, the proposed MF-Net achieves higher segmentation accuracy.

## Related Work

Recently, deep learning-based method has been proposed for image segmentation and achieved remarkable results. Long et al. (2015) proposed a fully convolutional networks (FCN) for semantic segmentation, which removed the full connection layer and could adapt to any input size. Although FCN has achieved satisfactory performance in semantic segmentation, the capacity of FCN to capture contextual information still needs to be improved as the limitation of convolutional layers. To tackle these problems, there are many methods that use pyramid-based modules or global pooling to aggregate regional or global contextual information (Chen et al., 2017; Zhao et al., 2017). Zhao et al. (2017) proposed a pyramid scene parsing network (PSPNet) based on pyramid pool modules, which aggregated context information from different regions to learn global context information. Chen et al. (2017) further proposed DeepLab v3 for semantic segmentation by introducing atrous convolution and atrous spatial pyramid pooling (ASPP). In addition, many attention mechanism-based methods have been explored to aggregate heterogeneous contextual information (Li et al., 2018; Oktay et al., 2018; Fu et al., 2019). However, these methods are mainly applied to the segmentation tasks with obvious features. In additional, there are also many deep learning-based methods have been proposed for medical image segmentation (Ronneberger et al., 2015; Gu et al., 2019; Feng et al., 2020). Although these methods have achieved impressive results, their performance of CNV segmentation in OCT images with large morphological differences, speckle noise, and other similar disease interference features has been reduced. Therefore, to improve the segmentation accuracy and tackle the challenges of CNV segmentation in retinal OCT images, a novel multi-scale information fusion network (MF-Net) is proposed for CNV segmentation in retinal OCT images.

## Method

**Figure 1:**
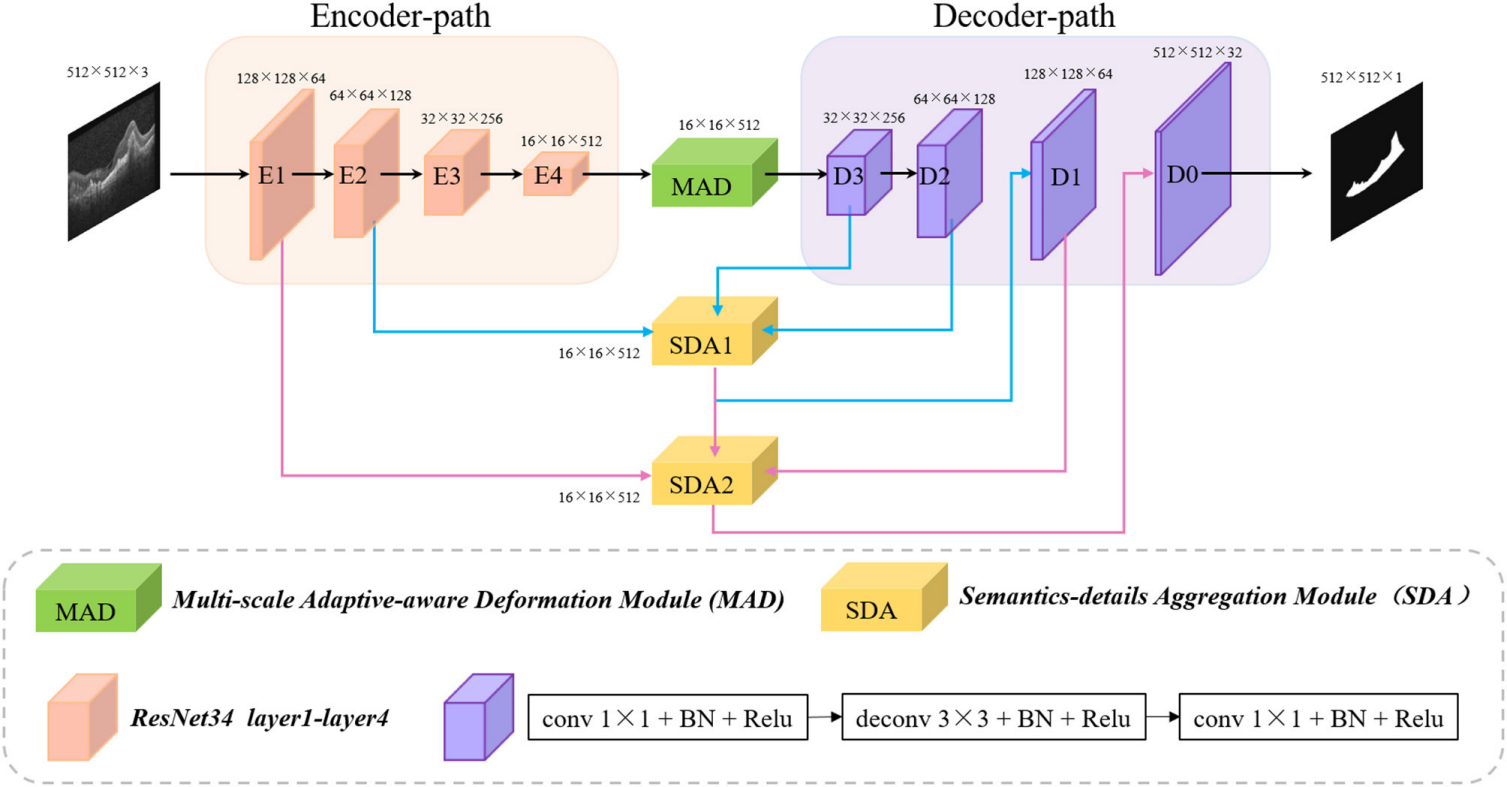
Architecture of the proposed MF-Net.

As shown in Figure 1, the proposed encoder-decoder structure-based multi-scale information fusion network (MF-Net) consists of three parts: encoder-decoder network, multi-scale adaptive-aware deformation module (MAD), and semantics-details aggregation module (SDA). Specifically, the encoder-decoder network is used as our backbone network. MAD is inserted at the top of the encoder to guide the model to focus on the multi-scale deformation maps and aggregate the contextual information, while SDA is applied as a variant of skip connection of the whole network to fuse multi-level semantic information.

### Backbone

Recently, the encoder-decoder structure is proved to be an efficient architecture for pixel-wised semantic segmentation. Most of the state-of-the-art segmentation networks are based on encoder-decoder structures, including AttUNet (Oktay et al., 2018), CE-Net (Gu et al., 2019), and PSPNet (Zhao et al., 2017) that have achieved remarkable performances in medical image segmentation. The encoder-path is mainly used to extract rich semantic information and global features from the input image and down sample the feature maps layer by layer, while the decoder-path aims to up sample the feature maps with strong semantic information from higher level stage, and restore the spatial resolution layer by layer.

To maximize the use of the information provided by the original image, the same encoder-decoder path is used as our backbone network. Unlike CE-Net, which send, the output of the encoder-path to dense atrous convolution (DAC) followed by residual multi-kernel pooling (RMP), the output is directly sent to the decoder-path. In addition, the skip-connection between the same level of encoder and decoder in CE-Net is also deleted in our backbone network.

### Multi-Scale Adaptive-Aware Deformation Module (MAD)

**Figure 2:**
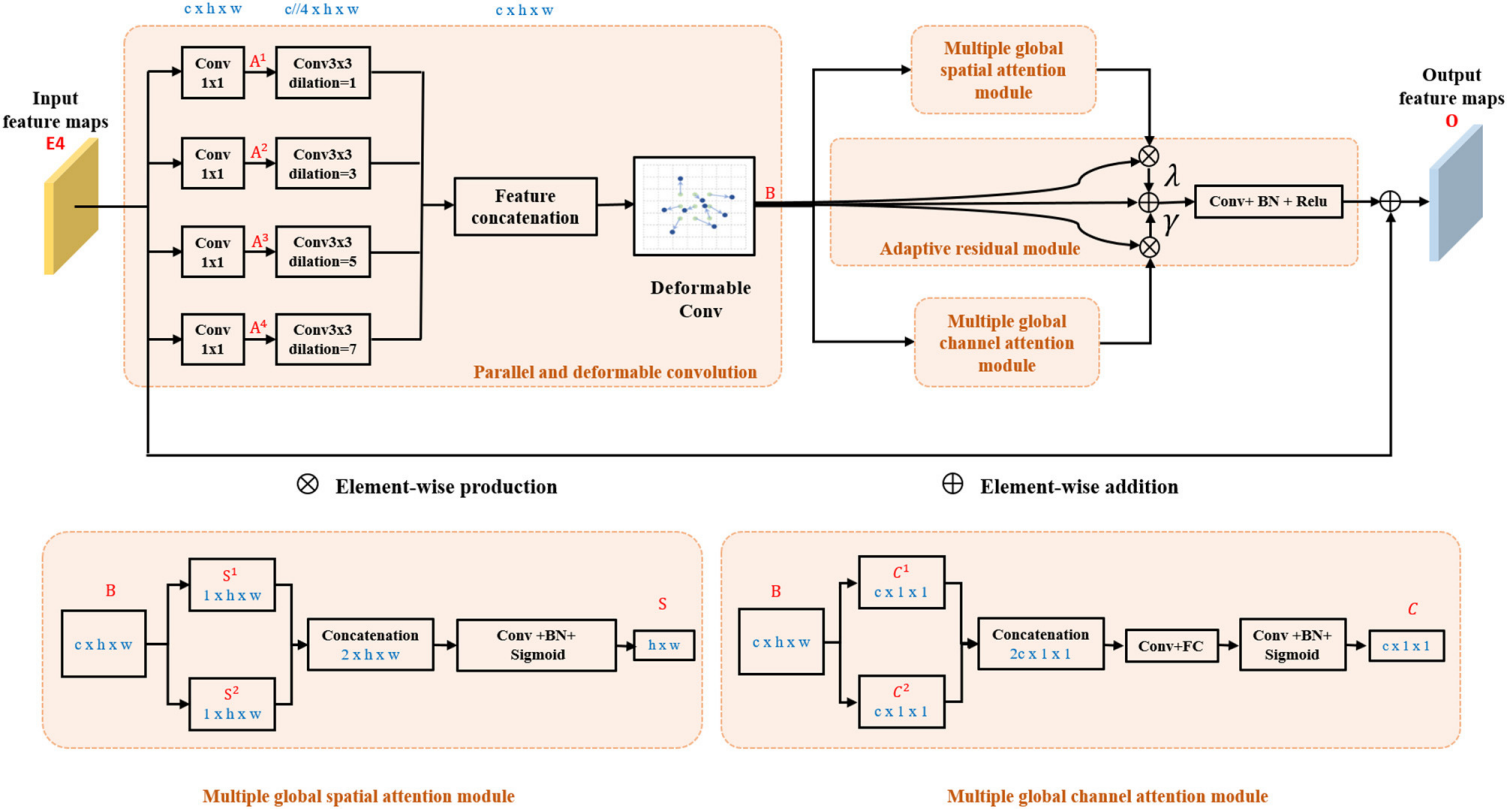
Architecture of the proposed multi-scale adaptive-aware deformation module (MAD).

It has been demonstrated that the multi-scale feature can improve the CNV segmentation accuracy in Zhang et al. (2019) and Xi et al. (2019). Therefore, to tackle the problems of large morphological differences of CNV in retinal OCT images, a MAD module is embedded at the top of the encoder-path to guide the model to focus on multi-scale deformation of the targets and aggregate the contextual information. As can be seen from Figure 2 that the MAD module contains four parts: parallel and deformable convolution module, multiple global spatial attention module, multiple global channel attention module, and adaptive residual module as shown in Figure 2.

#### Parallel and Deformable Convolution Module

After features are encoded by Encoder 4 (E4), they are fed into parallel and deformable convolution module to augment the spatial sampling locations in the modules by additional offsets of kernel size in horizontal and vertical direction. As shown in Figure 2, the output of Encoder 4 (E4) is simultaneously fed into four 1×1 convolutional layers. Four dilation convolutions with rate 1, 3, 5, and 7 are, respectively, further used after the four parallel layers to squeeze the channel and to extract global context information from different levels of feature maps, and then, the feature maps are concatenated and fed into a deformable convolution to compute *B* ∈ *R*^*c*×*h*×*w*^. Finally, *B* ∈ *R*^*c*×*h*×*w*^ are fed into the parallel-linked multiple global spatial attention module, multiple global channel attention module, and adaptive residual module, respectively. The parallel and deformable convolution module can be summarized as


(1) $$\begin{array}{l} B=Conv_{deform}concat_{k=1}^{4}\left(conv_{dilation}{@}_{2k-1}\left(A^{k}\right)\right), \end{array}$$

where *A*^*k*^ ∈ *R*^*c*×*h*×*w*^ denotes the output of 1×1 convolutional layers in *k*-th parallel branch, and @_2*k*−1_ represents the convolution with a dilation rate of 2*k* − 1.

#### Multiple Global Spatial Attention Module

Max-pooling and average pooling are commonly used operations in convolutional neural networks, since they can reduce the sizes of feature maps and keep significant spatial response information in each channel; nevertheless, noise may also be kept due to the different sizes and shapes of the lesion. To reduce the influence of the irrelevant significant spatial response information in all channels, average pooling can be used to compute the mean value of all channels in the corresponding position in the input feature maps. Therefore, 2D average-pooling and max-pooling are performed simultaneously in our multiple global spatial attention module to get the most significant spatial response information in all channels and suppress noise interference. *B* are fed to the maximum map branch and the mean map branch in parallel to generate attention map *S*^1^ ∈ *R*^1×*h*×*w*^ and *S*^2^ ∈ *R*^1×*h*×*w*^, respectively, and then are concatenated in channel dimension. Then, a convolutional operation is applied to squeeze the channel of concatenated maps. Finally, a sigmoid function is used to generate the final attention feature map *S* ∈ *R*^1×*h*×*w*^,


(2) $$\begin{array}{l} S=sigmoid\left(conv\left(concat\left(S^{1},S^{2}\right)\right)\right). \end{array}$$

This module can get the response of each feature map in all channels and suppress noise interference.

#### Multiple Global Channel Attention Module

Two parallel branches with global pooling are also constructed. The feature maps *B* are fed into a global max-pooling operation to obtain global channel maximum value maps *C*^1^ ∈ *R*^*c*×1×1^, while *B* are also fed into a global average-pooling operation to obtain global channel mean value maps *C*^2^ ∈ *R*^*c*×1×1^. Then, *C*^1^ and *C*^2^ are concatenated and fed into a convolution layer to smooth and squeeze the feature maps. Finally, the results are reshaped and fed into a fully connected layer followed by a sigmoid function to obtain the final feature map *C* ∈ *R*^*c*×1×1^,


(3) $$\begin{array}{l} C=sigmoid\left(FC\left(conv\left(concat\left(C^{1},C^{2}\right)\right)\right)\right). \end{array}$$

This module can get the response of each feature map in all channels and suppress noise interference.

#### Adaptive Residual Module

The output of parallel and deformable convolution module *B* ∈ *R*^*c*×*h*×*w*^ is multiplied by feature maps from multiple global spatial attention module *S* ∈ *R*^1×*h*×*w*^ spatial-wisely and feature maps from multiple global channel attention module *C* ∈ *R*^*c*×1×1^ channel-wisely, respectively. Then, pixel-wise addition operation followed by a convolutional layer is applied as


(4) $$\begin{array}{l} O=B⊕conv\left(\left(\lambda B{⊗}_{spatial}\left(S\right)\right)⊕\left(\gamma B{⊗}_{channel}\left(C\right)\right)\right), \end{array}$$

where ⊗_*spatial*_ and ⊗_*channel*_ denote spatial-wise and channel-wise multiple, respectively. *O* ∈ *R*^*c*×*h*×*w*^ represents the output of the adaptive residual module. ⊕ represents pixel-wise addition. λ and γ are learnable parameters and are initialized as a non-zero value (1.0 in this study). Finally, pixel-wise addition is used to add the original feature maps to the smoothed feature maps to get the final output of multi-scale adaptive-aware deformation module *O* ∈ *R*^*c*×*h*×*w*^ to the decoder-path.

### Semantics-Details Aggregation Module (SDA)

**Figure 3:**
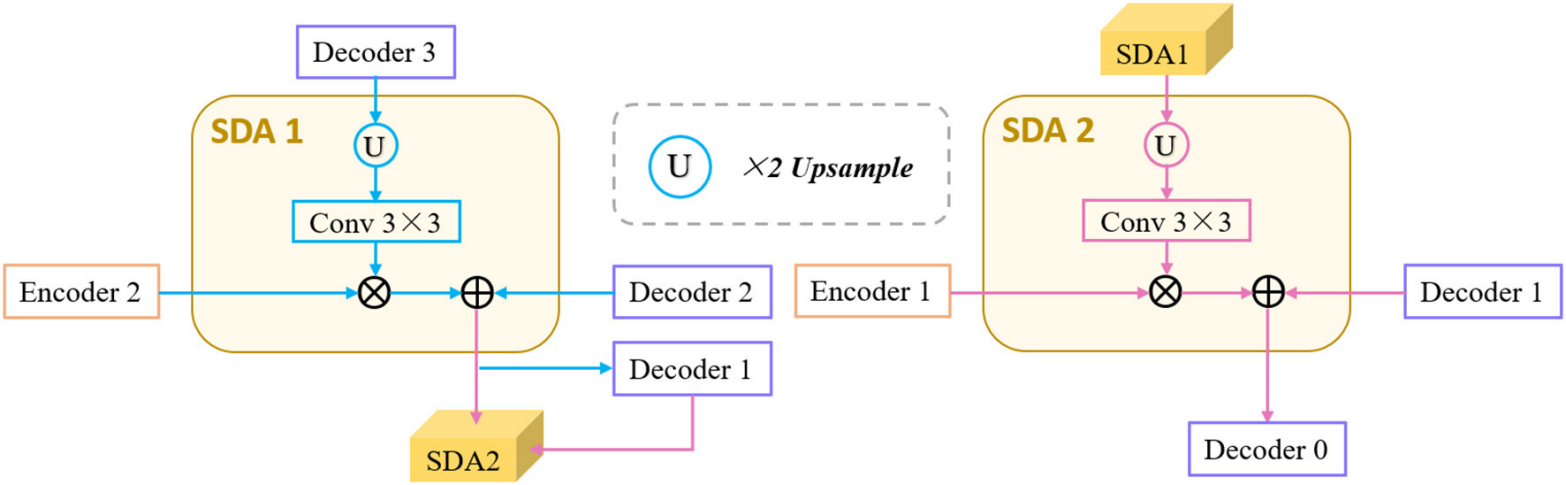
Architecture of the proposed semantics-details aggregation module (SDA).

Skip-connection can fuse the strong semantic information of the decoder-path with the high-resolution feature of the encoder-path. It is a commonly used structure in encoder-decoder-based network and further promotes the applications of the encoder-decoder structure. However, directly sending the high-resolution features of the encoder to the decoder will introduce irrelevant clutters and result in incorrect segmentation. Therefore, a novel semantics-details aggregation module (SDA) has been proposed as a variant of skip-connection to enhance the information that is conducive to segmentation and suppress invalid information. As can be seen in Figure 1, two SDA modules have been introduced between encoders and decoders. The structure of the proposed SDA module is shown in Figure 3. In the SDA module, the skip-connection is reconstructed by combining the feature map of encoder, decoder, and upper-level decoder. For example, the left of Figure 3 shows the structure of SDA 1. First, output feature maps of the Decoder 3 are upsampled followed by 3 × 3 convolutional layers to squeeze the channel. Then, the obtained feature maps and the output of the Encoder 2 are multiplied pixel-wisely to filter the detailed information that is conducive to segmentation. Finally, the filtered feature maps and the output of the Decoder 2 are added pixel-wisely to fuse detailed information and high-level semantic information. Above all, each SDA module in different stages can be summarized as


(5) $$\begin{array}{l} S^{k}=Conv\left(F^{k}{@}_{2}\right)⊗E^{3-k}⊕D^{3-k},k=1,2, \end{array}$$

where *S*^*k*^ denotes the output of the *k*-th SDA module, and @_2_ represents the upsampling operation with rate of 2. *E*^*k*^ and *D*^*k*^ denote the output feature maps of the *k*-th Encoder and Decoder. *F*^1^ and *F*^2^ represent the output feature maps of the Decoder 3 and SDA 1, respectively. *S*^*k*^ denotes the output of the *k*-th SDA module. It is worth noting that no skip-connection is introduced after Encoder 3 and Encoder 4, because the detailed information may be gradually weakened when transmitted to the deeper layers, and also, it can save computing resources.

### Loss Function

Image segmentation tasks can be analogized to pixel-level classification problems. Therefore, the binary cross-entropy loss *L*_*BCE*_, commonly used in classification tasks, is adopted to guide the optimization of our proposed method. However, *L*_*BCE*_ only be adopted to optimize segmentation performance in pixel level, ignoring the integrity of the image level. Therefore, to tackle this problem, the dice loss also be introduced to optimize our proposed method. The joint loss function as


(6) $$\begin{array}{l} L_{Real}=L_{Dice}+L_{BCE}, \end{array}$$

(7) $$\begin{array}{l} L_{Dice}=1-\sum_{h,w}\frac{2|X\times Y|}{\left|X|+|Y\right|}, \end{array}$$

(8) $$\begin{array}{l} L_{BCE}=-{\sum^{\text{​}}}_{h,w}\left(Y\;\log\;X+(1-Y)\;\log\;(1-X)\right), \end{array}$$

where *X* and *Y* denote the segmentation results and the corresponding ground truth, and *h* and *w* represent the coordinates of the pixel in *X* and *Y*.

### SemiMF-Net

**Figure 4:**
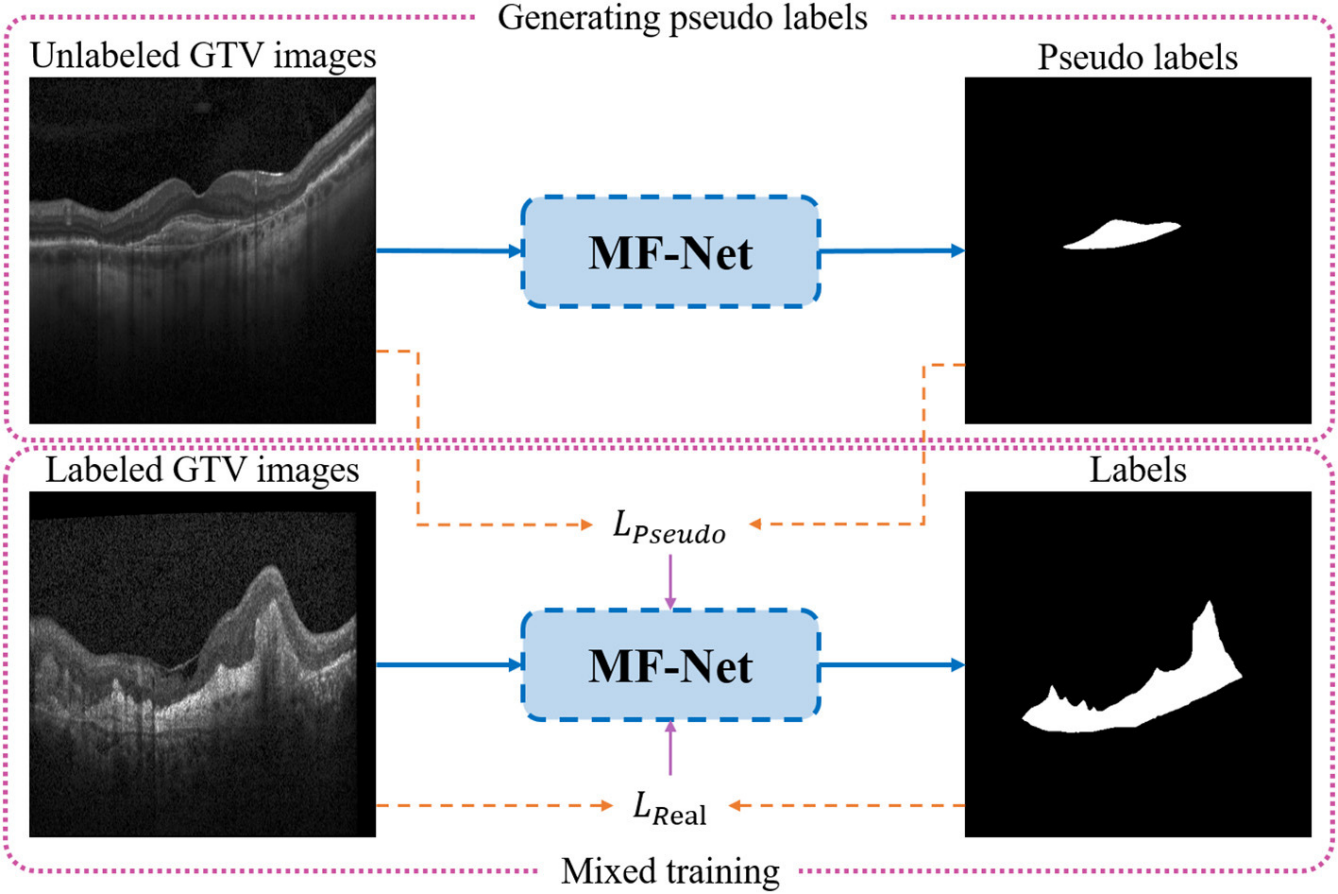
Architecture of the proposed SemiMF-Net.

In medical image segmentation tasks, the lack of pixel-level annotation data has always been one of the important factors that hinder the further improvement of segmentation accuracy, and it is expensive and time-consuming to obtain these label data. Therefore, it has always been an urgent problem in the field of medical image segmentation to use unlabeled data combined with limited labeled data to further improve segmentation performance. To this end, based on the newly proposed MF-Net, a novel SemiMF-Net is further proposed by combining the pseudo-label augmentation strategy to leverage unlabeled data to further improve the CNV segmentation accuracy, as shown in Figure 4. It can be seen from Figure 4 that our proposed semi-supervised framework of SemiMF-Net mainly consists of three steps: (1) Limited labeled data are adopted to pre-train MF-Net to segment unlabeled, and these segmentation results are employed as pseudo-labels for unlabeled data. (2) Unlabeled data with pseudo labels and labeled data are mixed to re-train the MF-Net based on the objective function *L*_*Pseudo*_ + β*L*_*Real*_ in a semi-supervised way, where *L*_*Pseudo*_ and *L*_*Real*_ are the joint loss function as Equation (6), and β is a weight paramter (1.0 in this study). (3) Finally, the SemiMF-Net that can accurately segment CNV in retinal OCT images is obtained.

## Experiments

### Dataset

In order to accurately segment CNV and evaluate the performance of the proposed method, experienced ophthalmologists annotate pixel-level ground truth for the 1,522 OCT images with CNV collected from the UCSD public dataset (Kermany et al., 2018), which collected by the Shiley Eye Institute of the University of California San Diego (UCSD) and all of the images (Spectralis OCT, Heidelberg Engineering, Germany) were selected from retrospective cohorts of adult patients without exclusion criteria based on age, gender, or race. In addition, to evaluate the performance of the proposed method and all comparison algorithms comprehensively and objectively, four-fold cross-validation is performed in all experiments, in which each fold contained 380 OCT images except the fourth fold that had 382 OCT images. In addition, 2,560 retinal OCT images from the remaining 35,683 OCT images are randomly selected as unlabeled data to participate in SemiMF-Net training. The details for data strategies are listed in Table 1.

**Table 1:** The details of data strategies.

	**Supervised**	**Semi-supervised**
Training	Retinal OCT images with ground truth from three folds.	Retinal OCT images with ground truth from three folds+2,560 retinal OCT images with pseudo labels.
Testing	Retinal OCT images with ground truth from the remaining one fold.	Retinal OCT images with ground truth from the remaining one fold.

### Implementation Details

Binary cross-entropy loss and dice loss are jointly used as the loss function to train the proposed network. The implementation of our proposed MF-Net is based on the public platform Pytorch and NVIDIA Tesla K40 GPU with 12GB memory. Adam is used as the optimizer. Initial learning rate is set to 0.0005, and weight decay is set to 0.0001. The batch size is set as 4 and epoch is 50. To be fair, all experiments adopt the same data preprocessing and training strategy.

### Evaluation Metrics

To comprehensively and fairly evaluate the segmentation performance of different methods, three indicators including dice similarity coefficients (DSC), sensitivity (SEN), and Jaccard similarity coefficient (JSC) are adopted to quantitatively analyze the experimental results, among which JSC and DSC are the most commonly used indices in validating the performance of segmentation algorithms (CE-Net, CPFNet, PSPNet, and DeepLabV3). In addition, the SEN is always adopted to evaluate the recall rate of abnormal conditions, which is essential for accurate screening of abnormal subjects and has been applied in many medical segmentation tasks (CE-Net, CPFNet, and AttUNet). The formulas of the three evaluation metrics are as follows


(9) $$\begin{array}{l} Dice=\frac{2TP}{FP+2TP+FN}, \end{array}$$

(10) $$\begin{array}{l} SEN=\frac{TP}{TP+FN}, \end{array}$$

(11) $$\begin{array}{l} JSC=\frac{TP}{FP+TP+FN}, \end{array}$$

where TP represents the number of true positives, FP represents the number of false positives, and FN represents the number of false negatives.

### Results

The proposed MF-Net and SemiMF-Net are compared with state-of-the-art methods, including UNet (Ronneberger et al., 2015), CE-Net (Gu et al., 2019), CPFNet (Feng et al., 2020), AttUNet (Oktay et al., 2018), DeepLab v3 (Li et al., 2018), and PSPNet (Chen et al., 2017), as shown in Table 2. Compared to the backbone, CE-Net achieves an increase of 0.21% for the main evaluation metric DSC, due to the combination of dense atrous convolution (DAC) and residual multi-kernel pooling (RMP). The performance of CPFNet is comparable with the proposed MF-Net as for the insertion of global pyramid guidance (GPG) module, which combines multi-stage global context information to reconstruct skip-connection and provide global information guidance flow for the decoder.

**Table 2:** The result of comparison experiments and ablation studies (mean ± SD).

**Methods**	**DSC**	**SEN**	**JSC**	**Time (s)**
UNet	92.38 ± 0.31	92.44 ± 0.97	85.92 ± 0.53	0.1158
CE-Net	92.73 ± 0.23	92.82 ± 0.81	86.52 ± 0.41	0.0921
CPFNet	92.77 ± 0.22	92.96 ± 0.52	86.58 ± 0.38	0.1053
AttUNet	92.31 ± 0.14	92.22 ± 0.37	85.81 ± 0.25	0.1289
DeepLabV3	92.73 ± 0.19	92.75 ± 0.25	86.55 ± 0.35	0.1316
PSPNet	92.62 ± 0.37	92.79 ± 0.29	86.32 ± 0.62	0.2237
Backbone	92.46 ± 0.29	92.56 ± 0.44	86.05 ± 0.50	0.0789
Backbone+MAD	92.71 ± 0.28	92.81 ± 0.39	86.48 ± 0.48	0.0842
Backbone+SDA	92.76 ± 0.18	92.69 ± 0.68	86.57 ± 0.33	0.0711
MF-Net	92.90 ± 0.21	93.01 ± 0.50	86.80 ± 0.37	0.0895
SemiMF-Net	93.07 ± 0.18	93.26 ± 0.45	87.07 ± 0.31	0.0895

It is worth noting that both proposed MF-Net and SemiMF-Net achieves better performance than all of the above methods. As shown in Table 2, the DSC, SEN, and JSC of MF-Net achieves 92.90, 93.01, and 86.80%, respectively. Compared to MF-Net, the average values of DSC, SEN, and JSC of the proposed SemiMF-Net have been improved to 93.07, 93.26, and 87.07%, respectively. These experimental results show that our proposed SemiMF-Net can leverage unlabeled data to further improve the segmentation performance.

It can be seen from Table 2 that our proposed method takes slightly longer time than backbone due to the introduction of MAD and SDA in MF-Net. However, it can still meet the requirement of real-time processing. These experimental results show that compared with other CNN-based methods, our proposed MF-Net and SemiMF-Net can achieve better segmentation performance with similar efficiency.

**Figure 5:**
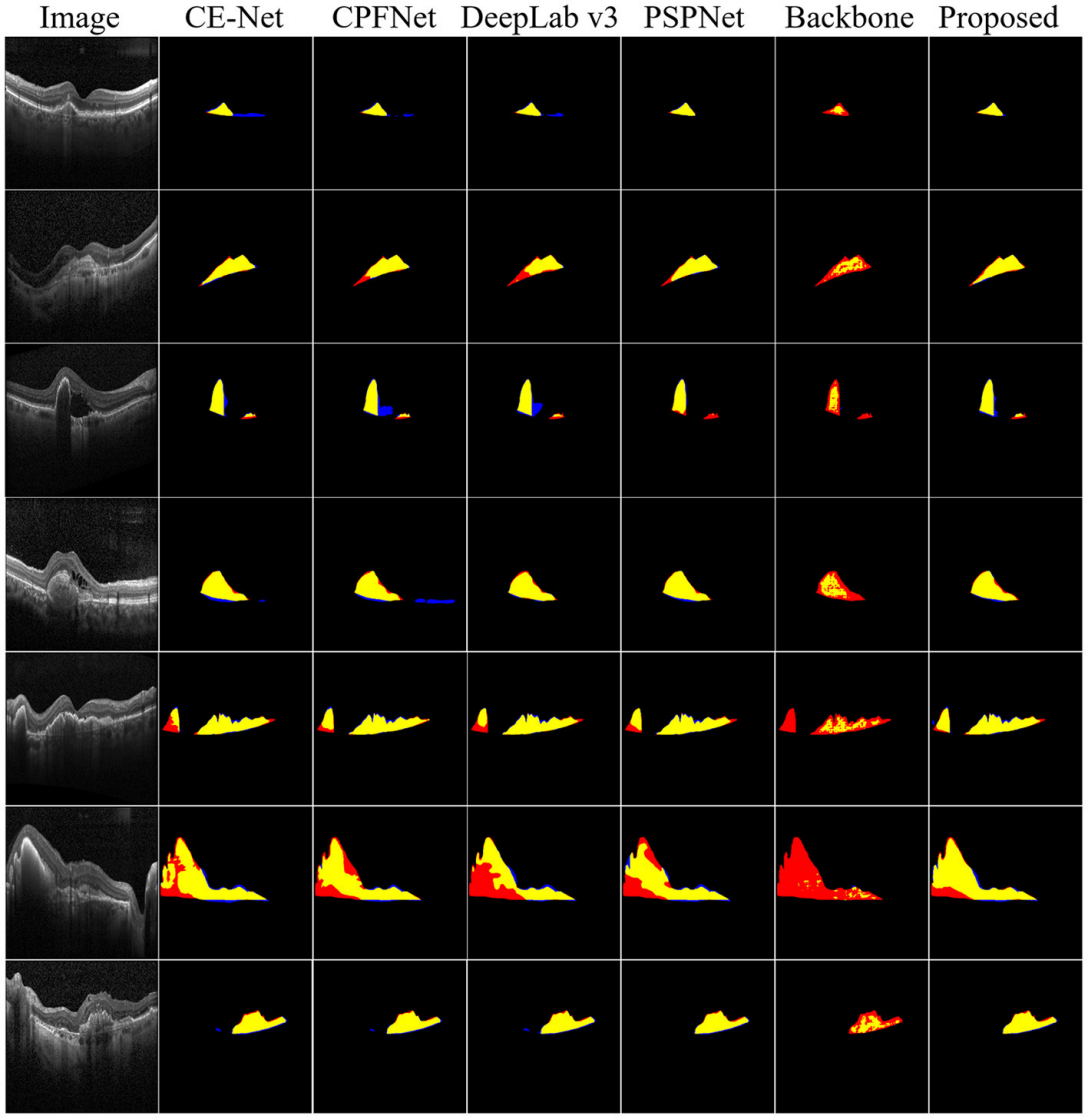
Examples of CNV segmentation. From left to right are original image, CE-Net, CPFNet, DeepLab v3, PSPNet, backbone, and our proposed method SemiMF-Net. Yellow represents the correctly segmented region, while red and blue are the results of false-positive segmentation and false-negative segmentation, respectively.

Furthermore, to demonstrate the effectiveness of the proposed method, the qualitative segmentation results are also given in Figure 5. The proposed SemiMF-Net is more accurate and has better robustness in the CNV segmentation task.

### Statistical Significance Assessment

We further investigate the statistical significance of the performance improvement for the proposed MF-Net and SemiMF-Net by the paired *t*-test, and these *p*-values are listed in Tables 3, 4, respectively.

**Table 3:** Statistical analysis (*p*-value) of the proposed MF-Net compared with other CNN-based methods.

**Method**	**JSC**	**DSC**
MF-Net-UNet (Ronneberger et al., 2015)	0.015	0.018
MF-Net-AttUNet (Oktay et al., 2018)	0.001	0.001
MF-Net-CE-Net (Gu et al., 2019)	0.001	<5E-4
MF-Net-PSPNet (Chen et al., 2017)	0.069	0.069
MF-Net-CPFNet (Feng et al., 2020)	0.004	0.003
MF-Net-DeepLab v3 (Li et al., 2018)	0.122	0.118
MF-Net-Backbone	0.002	0.002

**Table 4:** Statistical analysis (*p*-value) of the proposed SemiMF-Net compared with other CNN-based methods.

**Method**	**JSC**	**DSC**
SemiMF-Net-UNet (Ronneberger et al., 2015)	0.013	0.014
SemiMF-Net-AttUNet (Oktay et al., 2018)	<5E-4	<5E-4
SemiMF-Net-CE-Net (Gu et al., 2019)	0.011	0.009
SemiMF-Net-PSPNet (Chen et al., 2017)	0.042	0.040
SemiMF-Net-CPFNet (Feng et al., 2020)	0.005	0.004
SemiMF-Net-DeepLab v3 (Li et al., 2018)	0.051	0.041
SemiMF-Net-Backbone	0.007	0.007
SemiMF-Net- MF-Net	0.046	0.038

As shown in Table 3 that compared with other CNN-based methods, except for the significance compared with PSPNet and DeepLab v3 is not obvious, all the improvements for JSC and DSC of MF-Net are statistically significant with *p* < 0.05. The results further prove the effectiveness of the proposed MF-Net. Table 4 lists the *p*-values of the proposed SemiMF-Net compared with MF-Net and other CNN-based methods. All the improvements for JSC and DSC of SemiMF-Net are statistically significant with *p* < 0.05. The results further prove that the proposed SemiMF-Net can leverage unlabeled data to further improve the CNV performance significantly.

### Ablation Study

To verify the validity of the proposed MAD module and SDA module, we also conduct ablation experiments. As shown in Table 2, the embedding of MAD module (Baseline + MAD) achieves substantial improvement over the backbone in terms of all metric, which proves that multi-scale deformation features and adaptively aggregate contextual information are conducive for segmentation.

Furthermore, numerical results show that, the embedding of SDA (baseline + SDA) also contributes to the performance improvement, suggesting that well-designed skip connections can extract detailed information that is more conducive to segmentation, thereby improving the accuracy of segmentation. Especially, our proposed MAD module and SDA module can be easily introduced into other encoder-decoder network, which is our near future work. Furthermore, the proposed MF-Net achieves the highest DSC, and these results further demonstrate the effectiveness of our proposed method.

## Conclusion

Choroid neovascularization segmentation is a fundamental task in medical image analysis. In this study, we propose a novel encoder-decoder based multi-scale information fusion network named MF-Net. A multi-scale adaptive-aware deformation module (MAD) and a semantics-details aggregation module (SDA) are integrated to the encoder-decoder structure to fuse multi-scale contextual information and multi-level semantic information that is conducive to segmentation and further improve the segmentation performance. Furthermore, to solve the problem of insufficient pixel-level annotation data, based on the newly proposed MF-Net, SemiMF-Net is proposed by introducing semi-supervised learning to leverage unlabeled data to further improve the CNV segmentation accuracy. The comprehensive experimental results show that the segmentation performance of the proposed MF-Net and SemiMF-Net outperforms other state-of-the-art algorithms.

There is still a limitation on this study that the proposed MF-Net is designed based on the encoder-decoder structure, and cannot effectively prove its generalization on different backbone networks. In future work, we will extend the proposed MAD and SDA to various backbones to further prove its stability and versatility, and strive to reduce the number of parameters.

## Data Availability Statement

The datasets presented in this study can be found in online repositories. The names of the repository/repositories and accession number(s) can be found below: https://data.mendeley.com/datasets/rscbjbr9sj/2.

## Ethics Statement

The retinal OCT B-scans used in this paper are collected from the UCSD public dataset, it has been mentioned in the paper's section of “Experiments, Dataset”. All data in UCSD public dataset, Institutional Review Board (IRB)/Ethics Committee approvals were obtained. The work was conducted in a manner compliant with the United States Health Insurance Portability and Accountability Act (HIPAA) and was adherent to the tenets of the Declaration of Helsinki.

## Author Contributions

QM conceptualized and designed the study, wrote the first draft of the manuscript, and performed data analysis. LW, TW, MW, FS, WZ, ZC, and XC performed the experiments, collected and analyzed the data, and revised the manuscript. All authors contributed to the article and approved the submitted version.

## Funding

This study was supported part by the National Key R&D Program of China (2018YFA0701700) and part by the National Nature Science Foundation of China (61971298 and 81871352).

## Conflict of Interest

The authors declare that the research was conducted in the absence of any commercial or financial relationships that could be construed as a potential conflict of interest.

## Publisher's Note

All claims expressed in this article are solely those of the authors and do not necessarily represent those of their affiliated organizations, or those of the publisher, the editors and the reviewers. Any product that may be evaluated in this article, or claim that may be made by its manufacturer, is not guaranteed or endorsed by the publisher.
